# High-Performance Grape Disease Detection Method Using Multimodal Data and Parallel Activation Functions

**DOI:** 10.3390/plants13192720

**Published:** 2024-09-28

**Authors:** Ruiheng Li, Jiarui Liu, Binqin Shi, Hanyi Zhao, Yan Li, Xinran Zheng, Chao Peng, Chunli Lv

**Affiliations:** China Agricultural University, Beijing 100083, China

**Keywords:** grape disease detection, multimodal data integration, mobile device deployment, real-time disease detection, deep learning in agriculture

## Abstract

This paper introduces a novel deep learning model for grape disease detection that integrates multimodal data and parallel heterogeneous activation functions, significantly enhancing detection accuracy and robustness. Through experiments, the model demonstrated excellent performance in grape disease detection, achieving an accuracy of 91%, a precision of 93%, a recall of 90%, a mean average precision (mAP) of 91%, and 56 frames per second (FPS), outperforming traditional deep learning models such as YOLOv3, YOLOv5, DEtection TRansformer (DETR), TinySegformer, and Tranvolution-GAN. To meet the demands of rapid on-site detection, this study also developed a lightweight model for mobile devices, successfully deployed on the iPhone 15. Techniques such as structural pruning, quantization, and depthwise separable convolution were used to significantly reduce the model’s computational complexity and resource consumption, ensuring efficient operation and real-time performance. These achievements not only advance the development of smart agricultural technologies but also provide new technical solutions and practical tools for disease detection.

## 1. Introduction

Grapes are one of the extensively cultivated fruit trees worldwide [1,2], offering diverse varieties and significant economic value [3,4]. As a major producer of grapes and wine, China ranks at the forefront globally in terms of vineyard area and production [5]. In the Bayannur region of Inner Mongolia, the unique climatic conditions facilitate widespread grape cultivation, making it an integral part of local agricultural development [6,7]. However, the growth process of grapes is susceptible to various diseases, such as downy mildew and black rot [8,9], which not only affect the growth and quality of the fruit but also lead to significant economic losses. Therefore, the development of an effective grape disease detection system is crucial for enhancing yield and quality while minimizing losses.

Traditional grape disease detection methods rely primarily on manual visual inspection and the expertise of agricultural specialists. Although intuitive, this approach presents several limitations: first, manual detection is inefficient and fails to meet the needs of large-scale cultivation [10,11]; second, the accuracy of manual identification is highly dependent on individual experience and subjective judgment, leading to potential misdiagnoses; finally, as labor costs continue to rise, the expense of manual detection also increases [12].

With the rapid advancement of information technology, especially the application of computer vision, the use of image processing and machine learning for disease detection has become a research focus [13,14,15]. Anuja and colleagues [16] have advanced the use of SVM-based fruit quality grading systems, achieving accuracies of 77.24% (k-NN), 82.75% (SRC), 88.27% (ANN), and 95.72% (SVM) for systems implementing defect accuracy. Hemamalini et al. [17] employed machine learning methods such as KNN, SVM, and C4.5 to classify fruit photographs. These algorithms determine whether fruit is damaged, but their specificity and sensitivity require enhancement; Andrushia A Diana et al. [18] proposed a feature selection method based on artificial bee colony optimization (ABC) to explore the identification and classification of grape leaf diseases. This method initially uses cellular automata filters for image preprocessing and background subtraction for background removal. Furthermore, color, texture, and shape features are extracted from the input dataset. The ABC algorithm is utilized to select optimal features. Their experimental results demonstrate reliable accuracy compared to other methods. Math RajinderKumar M et al. [19] developed a deep convolutional neural network (DCNN) model to identify and classify grape diseases based on RGB leaf images, achieving an accuracy of 99.34% with precision, recall, and F1 scores of 0.9934. These results indicate the model’s capability to accurately identify and classify common grape diseases based on RGB leaf images. However, their model is computationally intensive, not easily deployable, and unable to provide real-time solutions from the agricultural domain.

In response, Zhang et al. [20] proposed a deep-learning-based YOLOv5-CA (Coordinate Attention) method to achieve an optimal balance between detection accuracy and speed in natural environments. This study integrates the CA mechanism into YOLOv5, highlighting visual features associated with downy mildew diseases and enhancing detection performance. Experimental results show that the proposed YOLOv5-CA achieves a detection accuracy of 85.59%, a recall rate of 83.70%, and mean average precision (mAP)@0.5% superior to common methods such as Faster R-CNN, YOLOv3, and YOLOv5. However, their model’s efficiency remains suboptimal, prompting Jin et al. [21] to introduce a novel architecture called GrapeGAN, which outperforms comparative models. Additionally, four convolutional neural network (CNN) recognition models are employed to identify generated grape leaf disease images. The results indicate that the recognition accuracy of grape leaf disease images generated by GrapeGAN exceeds 86.36%, with VGG16 and InceptionV1 achieving an accuracy of 96.13%. However, their model is specific to grapes and lacks general applicability; Sharma Vivek et al. [22] proposed a deeper lightweight convolutional neural network architecture (DLMC-Net) for plant leaf disease detection on multiple crops, suitable for real-time agricultural applications. In the proposed model, a series of collective blocks and channel layers are introduced to extract deep features. Additionally, pointwise convolution blocks and separable convolution blocks are employed to reduce the number of trainable parameters. The effectiveness of the proposed DLMC-Net model has been validated on four public datasets: citrus, cucumber, grapes, and tomatoes. Experiments show that the proposed model outperforms all considered models in terms of accuracy for citrus, cucumber, grapes, and tomatoes, with accuracies of 93.56%, 92.34%, 99.50%, and 96.56%, respectively. Thus, it can be asserted that the proposed model is a viable alternative for plant leaf disease detection across multiple crops.

This article addresses the implementation of an automated grape disease detection system based on computer vision and deep learning technologies in agricultural areas like Bayannur. Real-time images from vineyards are captured and analyzed using deep learning models, which accurately identify and classify grape diseases. Moreover, the proposed detection system is not confined to laboratory settings but is also designed for on-site deployment in actual vineyards, enabling large-scale real-time disease monitoring in conjunction with mobile devices. The main contributions of this paper are as follows:

- The use of parallel heterogeneous activation functions: Unlike traditional deep learning models that commonly employ a uniform type of activation function, such as ReLU or Sigmoid, which simplifies model design but limits the ability to process complex data, a novel network architecture employing parallel heterogeneous activation functions is proposed. This structure utilizes various activation functions simultaneously within the same network layer to accommodate different types of data features.
- Multimodal data fusion: A multimodal data fusion framework is developed by integrating data from various sensors. Traditional single-modal inputs often fall short in complex disease scenarios, whereas multimodal data fusion leverages the complementary nature of different sensor data, significantly enhancing the detection algorithm’s accuracy and adaptability to environmental conditions.
- Design of heterogeneous loss functions: A new heterogeneous loss function is designed within a multi-task learning framework to simultaneously optimize multiple output metrics of the model, such as classification accuracy and localization precision. This loss function integrates characteristics of both classification and regression losses, dynamically adjusting the weights of different tasks to achieve balanced optimization.
- Rapid on-site deployment: Tailored to the specific conditions of vineyards in the Bayannur area, a lightweight model deployment scheme is developed. Additionally, an adaptive algorithm is designed to dynamically adjust the computational load based on the actual operating conditions of the device, ensuring stable operation in the variable and complex field environment. This capability for rapid on-site deployment significantly enhances the practicality and widespread adoption of the disease detection system, providing robust technical support for grape production in the Bayannur region.

Through the comprehensive application of these technologies, the research not only improves the efficiency and accuracy of grape disease detection but also actualizes the technology, supporting the sustainable development of the grape industry in Bayannur and beyond.

## 2. Related Work

### 2.1. CNN Networks

CNNs represent a deep learning framework that excels in the field of image processing, widely applied to tasks such as image classification, object detection, and semantic segmentation [23,24,25]. The significant performance of CNNs in these areas is mainly attributed to their unique network architecture that effectively extracts and learns features automatically from images [26,27]. This characteristic is particularly crucial in the domain of agricultural disease detection, where the ability to accurately identify disease features from complex backgrounds is essential, a challenge that traditional methods often fail to meet [28,29].

A CNN is primarily composed of convolutional layers, pooling layers, and fully connected layers, each transforming the input data in specific ways, ultimately producing outputs usable for classification or regression tasks. In the convolutional layers, the network employs kernels (or filters) to extract local features from images, with each kernel responsible for extracting a specific type of feature, such as edges, colors, or textures. The convolution operation is mathematically represented as

(1) $$f\left(x,y\right)=\left(I*K\right)\left(x,y\right)=\sum_{m=-a}^{a}\sum_{n=-b}^{b}I\left(x+m,y+n\right)\cdot K\left(m,n\right)$$

where *I* represents the input image, *K* is the convolutional kernel, and f(x,y) is the output feature map, with (m,n) and (x,y) denoting the spatial positions in the kernel and output feature map, respectively. The pooling layer serves to reduce the spatial dimensions of the feature maps, thereby decreasing the computational demand and the risk of overfitting. Common pooling operations include max pooling and average pooling, formulated as follows:

(2) $$P\left(x,y\right)=\max_{(m,n)\in W}I\left(x+m,y+n\right)$$

where P(x,y) denotes the result after pooling, and *W* is the pooling window, with max indicating the maximum value within the window, helping preserve prominent features like edges. The fully connected layer maps the output features from preceding layers to the sample’s label space, typically used for generating final classification or regression outcomes. The operation of a fully connected layer can be viewed as a weighted summation process:

(3) y=σ(Wx+b)

where *x* is the input feature, *W* and *b* are the weight matrix and bias vector, respectively, and σ is the activation function, such as ReLU or Sigmoid, introduced to incorporate non-linearity, enabling the network to learn more complex patterns. In the field of grape disease detection, traditional manual feature extraction methods are not only inefficient but often struggle to adapt in variable field environments. In contrast, CNNs automatically learn the most effective features, greatly enhancing the accuracy and robustness of disease detection [30]. Architectures like AlexNet [31], VGGNet [32], and ResNet [33] have shown exceptional performance in plant disease detection tasks by increasing the network depth and width and optimizing network structures [34]. In particular, ResNet’s introduction of the residual learning mechanism alleviates the degradation problem during training:

(4) f(x)=H(x)+x

where *x* is the input feature and H(x) represents the residual mapping. This design allows effective training even in very deep networks, further enhancing disease detection performance.

However, as the scale of models increases, so does the computational complexity and resource consumption, posing challenges for deployment on resource-limited field devices [35,36]. To address these challenges, a grape disease detection network based on parallel heterogeneous activation functions is proposed in this article. This network structure enhances the model’s expressiveness and adaptability by using different activation functions in parallel. Moreover, to further increase the system’s practicality and deployability, multimodal data are incorporated, improving the accuracy and robustness of disease detection through multimodal fusion techniques. This fusion not only gathers complementary information from different physical properties but also enhances model performance in actual field conditions through optimized fusion strategies [37].

### 2.2. Multimodality

The application of multimodal technology in grape disease detection represents a solution to the limitations of single data sources in complex environments. By integrating data from diverse origins, multimodal systems can provide more stable and accurate detection results under various conditions [38,39,40]. The fundamental premise of multimodal technology is that different types of data, such as images, sounds, and texts, can offer complementary information, enabling higher processing performance and recognition accuracy than single data sources through appropriate fusion strategies [41]. In the context of grape disease detection, common data modalities include RGB images, infrared images, and spectral data. Each modality has its unique advantages: RGB images provide rich color and texture information [42]; infrared images capture hot spots related to plant health conditions [43]; and spectral data accurately reflect changes in plant biochemical components [44]. Multimodal data fusion typically occurs at three levels:

Data-level fusion involves directly merging raw data from different modalities into the model for processing. This method is straightforward but may face challenges due to the heterogeneity of the data. Feature-level fusion involves independently extracting features from each modality and then combining these features into a unified representation for further processing. This approach can be expressed by the following formula:

(5) $$F=\mathrm{Concat}(f_{1},f_{2},\ldots ,f_{n})$$

where *F* represents the fused feature vector, and $f_{1},f_{2},\ldots ,f_{n}$ are the feature vectors extracted from each modality, with Concat denoting the concatenation operation. Decision-level fusion occurs in the final stage of the model, where decision outcomes from each modality are combined to make the final judgment. This fusion typically uses a weighted average or voting mechanism, represented as

(6) $$D=\sum_{i=1}^{n}w_{i}\cdot d_{i}$$

where *D* is the final decision result, $d_{i}$ represents the decision result from each modality, and $w_{i}$ are the corresponding weight coefficients. In this research, a feature-level fusion strategy is adopted [45], combined with a network employing parallel heterogeneous activation functions for the efficient fusion of multimodal data. Additionally, an adaptive weight adjustment mechanism is introduced, allowing the influence of different modal features to be dynamically adjusted based on feedback during the training process. This adaptive mechanism not only enhances the flexibility of the model but also enables it to better adapt to different disease types and complex field environments.

In summary, by integrating multimodal data fusion with advanced network structures utilizing parallel heterogeneous activation functions, this research significantly improves the accuracy and robustness of grape disease detection. The application of this technology is not limited to grape disease detection; its principles and methods can be extended to other agricultural disease detections and environmental monitoring fields, demonstrating broad application potential and practical value.

## 3. Materials and Method

### 3.1. Dataset Collection

**Figure 1:**
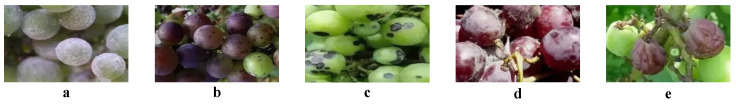
Dataset samples. (**a**) is powdery mildew; (**b**) is anthracnose; (**c**) is black rot; (**d**) is gray mold; (**e**) is white rot.

In this study, our dataset was collected from Bayannur City in Inner Mongolia and through internet channels, primarily to train and validate our proposed grape disease detection model. The dataset includes a large number of images of grape diseases, covering five main types of diseases: powdery mildew, anthracnose, black rot, gray mold, and white rot of grapes. The number of images collected ranges from 1400 to 1900 per disease type, with each type being collected in a balanced manner to ensure the comprehensiveness and fairness of the model training. The quantity of images collected for each disease is shown in Table 1 and Figure 1.

To meticulously record the characteristics of each grape disease, a detailed description was made for each type. For example, powdery mildew (Figure 1a) typically appears as a white powdery substance on the surfaces of leaves, young branches, and fruits; anthracnose (Figure 1b) is characterized by black or brown spots forming on fruits, leaves, and branches. Black rot (Figure 1c) manifests as circular black spots on the fruit, which can lead to cracking in severe cases; gray mold (Figure 1d) mainly occurs in humid environments, with a gray mold-like substance covering the fruit surface; white rot (Figure 1e) usually occurs on injured or cracked fruits, causing rapid decay of the fruit. Data collection was conducted in two main parts: field collection and online collection. Field collection primarily took place in vineyards, where, through cooperation with local farmers and agricultural technicians, we were able to obtain images of grapes at various stages of growth, both healthy and disease-affected. These images were later screened and annotated by professionals to ensure their accuracy and representativeness. Online collection involved gathering images from publicly available databases and related agricultural research websites, which were further verified and filtered to meet our research needs. In addition to image data, we also collected sensor data related to the grape growing environment, including parameters like infrared sensing, temperature, and humidity. These data were automatically recorded by sensors installed in the vineyards and then transmitted to our database via wireless networks. These environmental data provide important insights for analyzing the conditions and patterns of disease occurrence, aiding our model in more accurately identifying disease types under different environmental conditions.

### 3.2. Dataset Preprocessing

#### 3.2.1. Image Dataset Augmentation

Image data augmentation is an essential component of training deep learning models, particularly for tasks like grape disease detection, which require high generalization capabilities. By introducing diversified data during the training phase, image augmentation techniques effectively enhance the model’s adaptability and robustness to new and unseen environments. In this context, the approach incorporating parallel heterogeneous activation functions and multimodal data fusion adopted in this study leverages augmented image data to achieve superior disease detection performance. Given the variability in the natural growth conditions and photographic angles of grapes, it is crucial for the model to recognize disease images captured from various angles. Rotational and flipping transformations are employed to enhance the orientation diversity in the dataset. Mathematically, a rotational transformation can be represented as

(7) $$I^{\prime }\left(x^{\prime },y^{\prime }\right)=I\left(x\cos\theta -y\sin\theta ,x\sin\theta +y\cos\theta \right)$$

where (x,y) and $\left(x^{\prime },y^{\prime }\right)$ are the pixel coordinates before and after rotation, respectively, and θ is the angle of rotation. This transformation aids in training the model to recognize features that are invariant to specific orientations.

Moreover, to simulate varying distances between the observer and the disease, scaling and cropping are used to mimic the visual effects of these distance changes. Scaling is typically achieved by adjusting the image size, while cropping involves cutting out new image regions:

(8) $$I^{\prime }\left(x^{\prime },y^{\prime }\right)=I\left(sx+x_{0},sy+y_{0}\right)$$

where *s* is the scaling factor, and $\left(x_{0},y_{0}\right)$ is the starting point of the crop. Such manipulations not only enhance the model’s adaptability to size variations but also help reduce the model’s dependence on specific image regions, thereby improving its generalization capability. Adjusting image brightness, contrast, and saturation simulates different lighting conditions, which is particularly crucial for grape disease detection in outdoor environments as lighting directly affects image quality and model recognition:

(9) $$I^{\prime }\left(x,y\right)=\alpha I\left(x,y\right)+\beta$$

where α adjusts the contrast and β modifies the brightness. These adjustments enable the model to adapt to various lighting conditions, enhancing detection accuracy. Adding random noise to images is a common data augmentation technique that simulates potential damage during image transmission or processing:

(10) $$I^{\prime }\left(x,y\right)=I\left(x,y\right)+n\left(x,y\right)$$

where n(x,y) represents the noise function, typically modeled as Gaussian noise. This process enhances the model’s robustness to deteriorations in image quality. Through these augmentation techniques, each original image can generate multiple variants, significantly expanding the scale and diversity of the training dataset. This not only helps the model learn richer features but also significantly enhances its performance in practical applications, especially when combined with strategies involving parallel heterogeneous activation functions and multimodal data fusion, further improving the accuracy and robustness of the model proposed in this study for grape disease detection tasks.

#### 3.2.2. Sensors Dataset Preprocessing

Sensor data play a crucial role in the detection of grape diseases, especially when combined with image data for multimodal analysis. To ensure that sensor data can be effectively utilized by deep learning models, a series of precise preprocessing steps are undertaken. These steps not only enhance the quality of the data but also ensure better integration with image datasets, thereby improving the accuracy and efficiency of grape disease detection.

Initially, the preprocessing of sensor data begins with data cleaning, which is vital for identifying and addressing anomalies and errors in the data. These anomalies are often caused by sensor malfunctions, data transmission errors, or environmental interferences. For instance, a temperature sensor might record abnormal readings due to sudden environmental changes or equipment malfunctions. Dealing with these anomalies typically involves setting thresholds, standardizing data, and sometimes applying data smoothing techniques. Mathematically, anomaly handling can be modeled by calculating the mean and standard deviation of the data and then filtering out data points that deviate from the mean by several standard deviations:

(11) if|x−μ|>kσ,thenremovex

where *x* is an individual data point; μ and σ are the mean and standard deviation of the dataset, respectively; and *k* is a coefficient controlling sensitivity. Following data cleaning, feature extraction constitutes another critical step. Since raw time-series data often contain substantial redundant information, direct utilization can lead to inefficiencies and model overfitting. Therefore, selecting appropriate feature extraction methods is crucial for capturing key environmental variables. For instance, features such as the daily average, maximum, and minimum temperatures, as well as the rate of change in humidity, can be extracted from temperature and humidity sensor data:

(12) $$T_{\mathrm{avg}}=\frac{1}{n}\sum_{i=1}^{n}T_{i},\;T_{\max}=\max\left(T_{i}\right),\;T_{\min}=\min\left(T_{i}\right)$$

where $T_{i}$ represents the temperature reading at time point *i* and *n* is the total number of measurements within a selected time window. The normalization of data ensures numerical stability during model training, typically scaling the feature values to a uniform range, such as between 0 and 1. This is achieved using the following formula:

(13) $$x^{\prime }=\frac{x-\min(x)}{\max(x)-\min(x)}$$

where *x* is the original data and $x^{\prime }$ is the normalized data. This process reduces the impact of varying scales of features, enhancing the convergence speed and generalization capability of algorithms. Time window segmentation is an essential aspect of handling sensor data, especially for models that analyze time-series dependencies. Segmenting continuous data into multiple time windows, with each window’s data serving as an independent instance for model input, not only aids in capturing dynamic changes over time but also provides more precise local temporal features for model training and prediction:

(14) $$X_{\mathrm{window}}=\left\{x_{t},x_{t+1},\ldots ,x_{t+n-1}\right\}$$

where $X_{\mathrm{window}}$ represents the data set within a time window, $x_{t}$ is the data point at time *t*, and *n* is the size of the window. Through these comprehensive preprocessing steps, sensor data are not only transformed into a format suitable for deep learning models but also fully utilizes its inherent information. Combined with strategies involving parallel heterogeneous activation functions and multimodal data fusion, these steps significantly enhance the accuracy and efficiency of grape disease detection. The implementation of these preprocessing techniques provides high-quality input data for deep learning models, forming the foundation for achieving high-performance disease detection models.

### 3.3. Proposed Method

#### 3.3.1. Overall

**Figure 2:**
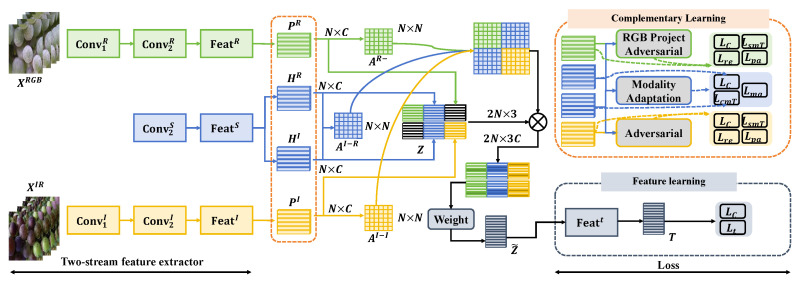
The figure shows the process of collecting input from different data sources, processing it through independent feature extraction paths, and then integrating and optimizing features through the parallel heterogeneous activation function module and the multimodal fusion module, finally outputting disease detection results. Each part’s specific functions and processes are detailed in the figure, demonstrating the model’s complexity and highly integrated processing capability.

This study presents a grape disease detection network based on multimodal data and parallel heterogeneous activation functions, designed to overcome the challenges of disease detection in complex agricultural environments. The network utilizes data from different sensors, including RGB images, infrared images, and spectral data, and processes and analyzes these data through a carefully designed network structure. The overall network architecture consists of several key components: data input, feature extraction, parallel heterogeneous activation modules, multimodal fusion, and output decision, as shown in Figure 2.

Each modality’s data are first processed through independent feature extraction channels, each including multiple convolutional layers (Conv), followed by activation functions to extract key features of that modality:

1. Feature extraction module: Specialized feature extraction pathways are set up for RGB images, infrared images, and spectral data. Each pathway includes two main Conv1 and Conv2, which are specifically adjusted to suit the characteristics of each data type.
  - The RGB data channel ($X_{RGB}$) includes successive convolutional layers Conv1*R* and Conv2*R*, followed by the feature layer Feat*R*, ultimately producing output $P_{R}$.
  - The infrared image data channel ($X_{IR}$) is similarly processed through Conv1*I* and Conv2*I*, outputting to the feature layer Feat*I*, resulting in $P_{I}$.
  - The spectral data channel is processed through a similar structure, outputting to the feature layer Feat*S*, resulting in $P_{S}$.
2. Parallel heterogeneous activation function module: Different activation functions such as ReLU, LeakyReLU, and PReLU are used in parallel after each convolutional layer, allowing the network to choose the most suitable activation method for different types of features within the same layer, thus enhancing the model’s ability to express features.
3. Multimodal fusion module: Features extracted from three independent data channels are uniformly processed by the fusion module. This module utilizes feature-level fusion techniques, such as feature vector concatenation (Concat), to combine all features into a comprehensive feature vector $Feat_{t}$, providing comprehensive information for subsequent decisions.
4. Output decision: The merged feature vector is passed to the decision layer, which combines information from all modalities, forming the final disease detection result through a weighted average or voting mechanism. This process involves complex weight adjustments and optimization to ensure high accuracy and robustness.

To optimize this network, a heterogeneous loss function is used, combining classification loss and regression loss, as well as a weight adjustment mechanism specifically designed for multitasking. This mechanism allows the model to adaptively adjust the weights of various tasks during the training process, optimizing the learning effects of different tasks. Through this design, the network can not only effectively process data from different sensors but also significantly enhance the performance of grape disease detection through the use of parallel heterogeneous activation functions and multimodal fusion technologies, providing strong technical support for intelligent disease management in complex agricultural environments.

#### 3.3.2. Parallel Heterogeneous Activation Function Module

**Figure 3:**
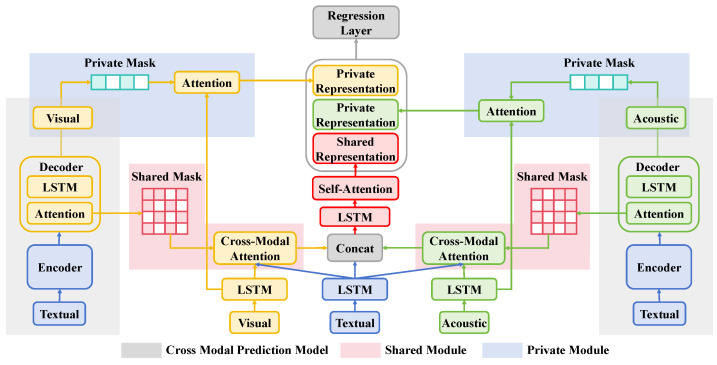
The structural schematic of the parallel heterogeneous activation function module proposed in this paper displays the configuration where different activation functions, such as ReLU, LeakyReLU, and PReLU, work in parallel in the same network layer and how they enhance the model’s capability to process various data features through a specific structure.

In the multimodal parallel heterogeneous activation function disease detection network proposed in this study, a core component is the parallel heterogeneous activation function module. This module is designed to utilize multiple activation functions within the same network layer to process different features, thereby enhancing the network’s adaptability and recognition capabilities across various data characteristics, as shown in Figure 3. This design not only improves the model’s precision in processing input data but also enhances its robustness in complex environments.

The parallel heterogeneous activation function module includes several convolutional layers, each followed by different activation functions that process the output of the same layer in parallel. Specifically, the module initially conducts feature extraction on the input data through a convolutional layer, and then the resulting feature map is simultaneously fed into multiple independent activation function channels. Each of these channels employs a different activation function, such as ReLU, LeakyReLU, and PReLU, to optimally process different types of data features. For instance, the ReLU activation function excels at processing positive signals, effectively transmitting activation signals; LeakyReLU performs better with signals containing negative values, preventing the excessive suppression of negative signals; and PReLU offers a parametrized slope, making the activation function response more flexible and adaptable to the specific data distribution.

The design of the module ensures that each activation function can independently learn the feature types best suited for its processing, and then these independently processed features are integrated through a fusion layer. The fusion layer uses concatenation to combine the feature maps output by each activation function into a comprehensive feature representation. This not only increases the network’s expressive power but also allows subsequent layers to learn useful information from a richer set of features. Mathematically, assuming the feature map output from the convolutional layer is *Z*, the outputs processed by each activation function are given as

(15) $$A_{ReLU}\left(Z\right)=\max\left(0,Z\right)$$

(16) $$A_{LeakyReLU}\left(Z\right)=\left\{\begin{array}{cc} Z & \mathrm{if}\;Z>0\\ 0.01Z & \mathrm{otherwise} \end{array}\right.$$

(17) $$A_{PReLU}\left(Z\right)=\left\{\begin{array}{cc} Z & \mathrm{if}\;Z>0\\ \alpha Z & \mathrm{otherwise} \end{array}\right.$$

where α is a learned parameter. Through this design, the model not only improves identification accuracy without sacrificing computational efficiency but also maintains high stability in variable field environments. Furthermore, by dynamically adjusting the weights or parameters of different activation functions, this parallel heterogeneous activation function module can further optimize the overall performance of the network, making it better adapted to different application scenarios and data variations, thereby playing a crucial role in complex tasks such as grape disease detection.

#### 3.3.3. Multimodal Fusion Module

**Figure 4:**
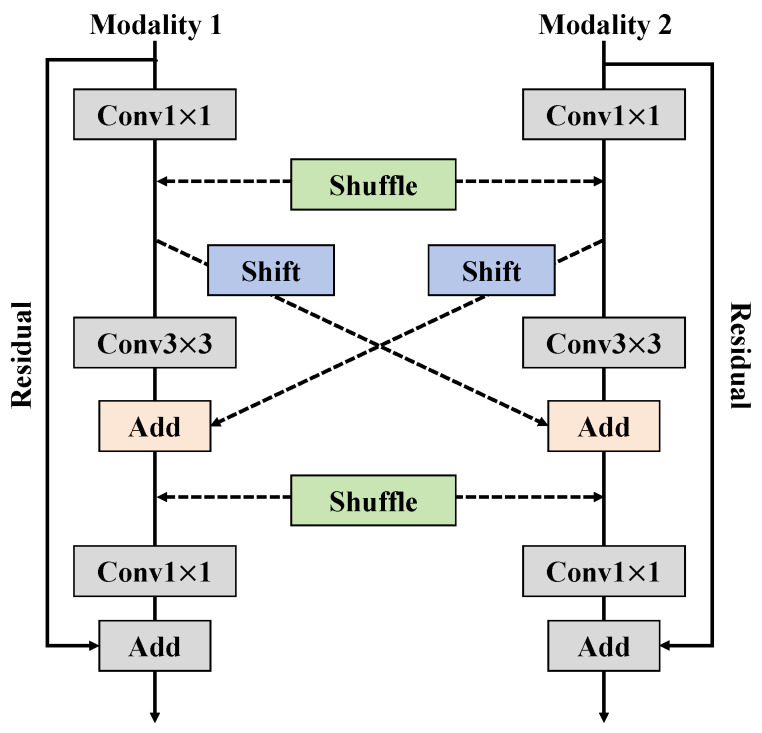
The structural schematic of the multimodal fusion module, which processes data from different sensors using specific convolution operations and residual connections, utilizing the shuffle and shift techniques to effectively merge features, enhancing the model’s accuracy and robustness in detecting grape diseases.

In this study, the multimodal fusion module is designed as a key component for integrating features from different sensors to enhance the accuracy and robustness of grape disease detection. The architecture of this module draws on the concept of the self-attention mechanism from the Transformer, but it has been significantly modified to accommodate the characteristics of multimodal data, as shown in Figure 4.

In traditional Transformer models, the self-attention mechanism allows the model to focus on the relationships within different parts of a sequence during data processing, primarily by calculating attention scores among elements in the sequence. In contrast, the multimodal fusion module focuses on effectively integrating information from diverse data sources. Unlike the Transformer’s self-attention, which deals only with relationships within a single modality, the multimodal fusion module is designed to integrate information across modalities, handling data with varied physical and statistical properties. The module includes several key steps: feature extraction, feature transformation, feature mixing, and residual connections. The specific processes are as follows:

1. Feature extraction: Data from each modality first undergo preliminary feature extraction through independent Conv1×1 and Conv3×3. This step aims to extract useful information from each modality and transform it into a higher-level feature representation.
2. Feature transformation: The initially extracted features are further transformed through another set of 1×1 convolution layers, preparing them for cross-modal feature mixing.
3. Feature mixing: The transformed features are then mixed using special shuffle and shift operations. The shuffle operation ensures that features from different modalities are spatially rearranged to enhance inter-feature interactions; the shift operation further enhances the model’s ability to integrate features.
4. Residual connections: To enhance model training stability and prevent gradient vanishing issues, residual connections are added after each processing step. These connections help the model maintain performance while increasing its depth.

Mathematically, this fusion process can be represented as

(18) $${\mathrm{Feat}}_{\mathrm{fused}}=\mathrm{Shift}\left(\mathrm{Shuffle}\left(\mathrm{Conv}1\times 1\left(\mathrm{ReLU}\left(\mathrm{Conv}3\times 3\left(\mathrm{Conv}1\times 1\left(F_{m}\right)\right)\right)\right)\right)\right)$$

where $F_{m}$ represents the input features from modality *m*, and the entire process includes convolution operations, ReLU activation, and shuffle and shift operations. The primary advantage of designing the multimodal fusion module is its ability to effectively merge information from different sensors, providing more comprehensive data analysis than any single modality alone. This is particularly important in grape disease detection tasks, as different types of sensor data, such as RGB images, infrared images, and spectral data, each have their strengths and limitations. Through multimodal fusion, the model can not only utilize the rich color and texture information provided by RGB images but also combine it with additional physiological and chemical information from infrared and spectral data to achieve more accurate and comprehensive disease diagnosis. Moreover, by employing mixing operations and residual connections, the fusion module maintains high training efficiency and stability without sacrificing the model’s depth and complexity. This design ensures superior performance during training and excellent adaptability during field deployment, which is crucial for the real-time monitoring and management of diseases in vineyards. Timely intervention facilitated by this model helps ensure the healthy growth and yield of crops.

#### 3.3.4. Heterogeneous Loss Function

In this study, a novel heterogeneous loss function was designed to more effectively handle multimodal data and enhance the accuracy of disease detection. This type of loss function significantly differs in structure and functionality from traditional loss functions and is particularly suitable for handling complex multi-task learning challenges, such as simultaneously classifying disease types and regressing disease locations in grape disease detection. Traditional loss functions are typically designed for a single task, such as cross-entropy loss for classification tasks or mean squared error loss for regression tasks. While these loss functions perform well when addressing a single task, they often fail to adequately balance the learning needs of multiple tasks in multi-task learning scenarios, potentially leading to suboptimal performance on some tasks. In contrast, the heterogeneous loss function combines multiple loss computation methods, allowing the model to balance and optimize different tasks during the training process, thus achieving better performance in a multi-task learning environment.

The heterogeneous loss function includes not only multiple types of task-specific loss calculations but also introduces a dynamic weight adjustment mechanism. This mechanism allows the loss function to adaptively adjust the impact weights of different tasks based on feedback during the training process, making the design more flexible and optimizing the overall model performance. The design of the heterogeneous loss function encompasses three parts: classification loss, regression loss, and fusion loss, each targeting specific task requirements. The mathematical expression for this is as follows:

(19) $$\mathrm{Loss}=\lambda_{1}\cdot L_{\mathrm{class}}+\lambda_{2}\cdot L_{\mathrm{reg}}+\lambda_{3}\cdot L_{\mathrm{fusion}}$$

Here, $L_{\mathrm{class}}$ represents the classification loss, assessing the model’s performance in disease type classification; $L_{\mathrm{reg}}$ represents the regression loss, evaluating the accuracy of disease location identification; $L_{\mathrm{fusion}}$ is the fusion loss, assessing the effectiveness of multimodal data fusion. The coefficients $\lambda_{1}$, $\lambda_{2}$, and $\lambda_{3}$ are weights that can be adjusted based on the importance of the respective tasks to optimize the model’s performance on each task.

The design of the heterogeneous loss function is grounded in optimization theory, aiming to minimize the overall loss while considering the importance and complexity of different tasks. By adjusting the weight coefficients, fine control over the model’s training process can be achieved, allowing the model to reach a good balance across various tasks. Mathematically, this weight adjustment can be considered a constrained optimization problem, which can be solved using the Lagrange multiplier method or other optimization algorithms to ensure that the loss is minimized under specific constraints.

In the application of grape disease detection, the heterogeneous loss function enables the model not only to accurately identify disease types but also to precisely locate the occurrence of diseases and to effectively utilize fused data from different sensors. The advantage of this approach lies in its provision of a systemic solution that comprehensively considers classification accuracy, localization precision, and data fusion effectiveness, significantly enhancing the overall performance of disease detection.

### 3.4. Evaluation Metrics

To comprehensively assess the performance of the grape disease detection system based on parallel heterogeneous activation functions and multimodality, various metrics were employed, including accuracy, precision, recall, mAP, and frames per second (FPS). These metrics not only evaluate the model’s performance in disease detection tasks but also reflect the response speed and processing efficiency of the model in practical applications. Accuracy is a standard metric that measures the proportion of correctly predicted samples out of the total samples. Precision assesses the proportion of true disease instances among the predicted disease samples, where higher precision indicates higher reliability of the model in disease prediction with fewer false positives. Recall measures the proportion of actual disease instances that were correctly identified, reflecting the model’s ability to capture disease presence; a higher recall indicates that the model can effectively recognize actual disease instances, thereby avoiding missed detections.

The mAP is used to measure the model’s precision at different recall levels, commonly employed in the domain of object detection, providing a comprehensive reflection of the model’s performance across various thresholds. The calculation of mAP involves plotting a precision–recall curve and then calculating the area under the curve:

(20) $$\mathrm{mAP}=\int_{0}^{1}p\left(r\right)\;dr$$

where p(r) represents the precision at recall *r*. FPS, an indicator of the model’s processing speed, reflects the number of frames the model can process per second, crucial for real-time disease detection systems. It directly impacts the user experience and practicality of the system in real deployments:

(21) $$\mathrm{FPS}=\frac{1}{T}$$

where *T* is the average time required by the model to process a single frame.

### 3.5. Baseline

In the evaluation of the grape disease detection system that incorporates parallel heterogeneous activation functions and multimodality, several state-of-the-art deep learning models in the domain of object detection and image processing were selected as benchmarks, including YOLOv3 [46], YOLOv5 [47], TinySegformer [48], DEtection TRansformer (DETR) [49], and Tranvolution-GAN. YOLOv3 and YOLOv5, popular models in real-time object detection systems, base their design on predicting bounding boxes and class probabilities using a single neural network. YOLOv3 incorporates multi-scale predictions and Darknet-53 as the feature extractor, featuring outputs at three different scales to predict objects of various sizes. YOLOv5, as the latest iteration in the YOLO series, further optimizes the model structure and inference speed by employing the CSPNet architecture, enhancing network flexibility and light-weighting, thus maintaining high accuracy while increasing processing speed. Following this, TinySegformer and DETR represent advanced methods based on Transformer for object detection. TinySegformer is designed as a lightweight Transformer aimed at segmentation tasks, minimizing computational demands. DETR utilizes the global attention mechanism of Transformers to predict objects and their bounding boxes directly. Lastly, Tranvolution-GAN combines the strengths of GANs (Generative Adversarial Networks) and Transformers, utilized for generating high-quality images by enhancing the realism and diversity of generated images while leveraging the Transformer to handle long-range dependencies.

Comparison with these models not only validates the effectiveness of the grape disease detection system employing parallel heterogeneous activation functions and multimodality but also provides deep insights into the model’s advantages and limitations in practical application scenarios. This comparative analysis aids in further optimizing the model structure and improving the accuracy and efficiency of disease detection, ultimately facilitating its application in actual agricultural production.

### 3.6. Experimental Setup

The experiments were primarily conducted on a computing platform equipped with high-performance GPUs, specifically using servers with NVIDIA Tesla V100 GPUs, which feature 32 GB of memory, sufficient to support large datasets and complex model training. The servers were also equipped with 256 GB of RAM and multicore Intel Xeon CPUs, ensuring efficient data processing and model training. On the software side, all experiments were conducted on Linux operating systems using Python programming, predominantly relying on the PyTorch deep learning framework. PyTorch provides a flexible development environment for deep learning, supporting automatic differentiation systems and various optimization algorithms, suitable for complex model design and experimentation. Additionally, CUDA and cuDNN libraries were utilized to optimize GPU computing performance.

During model training, a series of strategies were employed to optimize training outcomes and enhance the model’s generalization capabilities. Specifically, cross-validation was implemented, detailed as five-fold cross-validation. By randomly dividing the dataset into five non-overlapping subsets, training with four subsets and testing with the remaining one, and rotating this process five times, a comprehensive assessment of the model’s performance on different data was achieved, reducing the influence of random errors. To prevent overfitting, an early stopping strategy was also employed; specifically, training would cease if no significant improvement in performance on the validation set was observed over 20 consecutive training epochs. This strategy effectively prevents the model from overfitting on training data while neglecting generalization performance.

Training parameters were set as follows: the initial learning rate was set at 0.001, using the Adam optimizer, which adjusts the learning rate automatically to accommodate the training process. The batch size was set at 32, balancing training efficiency and accommodating the memory capacity of the GPU. To ensure the reliability of the results and facilitate subsequent analysis, detailed records were kept of the model’s training time, loss values per training epoch, and performance metrics on the validation set. These data not only help monitor the training process and analyze trends in model performance but also serve as a basis for adjusting the model structure and parameters.

## 4. Results and Discussion

### 4.1. Disease Detection Results

This study designed an experiment to validate and evaluate the performance of the proposed model in grape disease detection relative to existing technologies. The experiment systematically showcases the detection effectiveness of various models by comparing five key performance metrics—precision, recall, accuracy, mAP, and FPS—as shown in Table 2.

The results for several different models are displayed in the table. The YOLOv3 model achieved a precision of 0.80, an recall of 0.77, an accuracy of 0.78, and an mAP of 0.79, processing 21 frames per second. This shows its good real-time processing capability, though its recognition accuracy needs improvement. YOLOv5 exhibited higher efficiency and accuracy than YOLOv3, with a precision, recall, and accuracy of 0.83, 0.80, and 0.81, respectively, and an mAP of 0.81, at a processing speed of 28 FPS, indicating its enhanced recognition performance while maintaining a high speed. The DETR model further improved all performance indicators, with a precision of 0.85, a recall of 0.82, an accuracy of 0.83, and an mAP of 0.84, processing 35 frames per second, using the Transformer as its main architecture to enhance model’s recognition ability through global feature understanding. The TinySegformer model showed even more significant improvements in these metrics, achieving a precision of 0.88 and a recall of 0.85, within accuracy and mAP of 0.86, and 42 FPS, demonstrating its effectiveness and accuracy in segmenting and detecting grape diseases. Tranvolution-GAN performed the best among all models, with a precision of 0.90, a recall of 0.87, an accuracy of 0.88, and an mAP of 0.89, with the highest FPS of 49, combining Generative Adversarial Network and Transformer technologies to significantly enhance image processing quality and speed. Our method exhibited the best performance on these metrics, with a precision of 0.93, a recall of 0.90, an accuracy of 0.91, and an mAP of 0.91, at 56 FPS. This exceptional performance benefits from the proposed multimodal parallel heterogeneous activation function network structure, which effectively integrates information from different sensors and optimizes the feature extraction and fusion process through the heterogeneous activation function module. Additionally, by introducing a dynamic adjustment mechanism within the network, the adaptability and robustness of the network to different disease features are enhanced, maintaining high efficiency and accuracy in detection performance under variable real-world conditions.

Overall, these experimental results validate the effectiveness of our method in enhancing the accuracy and real-time capabilities of grape disease detection. By comparing with other advanced models, it is evident that our method has advantages in handling complex and variable agricultural image data, particularly in efficiently integrating multimodal data and enhancing computational performance. These achievements not only advance grape disease detection technology but also provide valuable references and application prospects for other agricultural disease detection tasks.

### 4.2. Ablation Experiment on Activation Functions

This experiment was designed to investigate the impact of different activation functions on the performance of the grape disease detection model. Through this experiment, the performance differences of various activation functions such as Tanh, ReLU, LeakyReLU, Sigmoid, and Mish under the same network architecture were systematically evaluated, as shown in Table 3. This method not only helps us understand the characteristics of each activation function but also provides data support for optimal network configurations.

The results indicate that the application of single activation functions like Tanh, ReLU, LeakyReLU, Sigmoid, and Mish has significantly different impacts on model performance. Models using the Tanh activation function achieved a precision of 0.75, a recall of 0.73, an accuracy of 0.74, and an mAP of 0.74, showing a certain baseline performance. When using the ReLU activation function, the model performance slightly declined, with a precision of 0.73, arecall of 0.71, an accuracy of 0.72, and an mAP of 0.72. The use of LeakyReLU slightly improved model precision to 0.76, with a recall of 0.73, an accuracy of 0.74, and an mAP of 0.75. The Sigmoid and Mish activation functions provided a higher precision, reaching 0.78 and 0.77, respectively, with recalls of 0.75 and 0.73, and correspondingly higher accuracy and mAP, demonstrating their advantages in handling certain specific features. The results of combining different activation functions were more prominent, showing the complementary effects of activation functions. For example, models combining ReLU, LeakyReLU, Sigmoid, and Mish achieved the best results among all experiments, with a precision of 0.93, recall of 0.90, accuracy of 0.91, and mAP of 0.91. This indicates that integrating the advantages of different activation functions can significantly improve model performance. Theoretically, the impact of different activation functions on model performance can be attributed to their mathematical characteristics. Tanh and Sigmoid activation functions output bounded activation values, suitable for scenarios requiring the suppression of extreme activations, but are prone to gradient vanishing, thus affecting the depth and efficiency of model training. The ReLU activation function provides non-linearity while maintaining gradient stability, suitable for deep networks, but has issues with dying neurons when handling negative values. LeakyReLU and Mish improve upon this by introducing a small negative slope or adaptive parameter adjustments, enhancing the model’s information utilization rate and robustness. Overall, selecting the appropriate activation function or their combination is crucial for optimizing the grape disease detection model. Experimental verification shows that combining multiple activation functions can effectively enhance the overall performance of the model, which is significant for handling complex disease detection tasks in practical applications. These findings not only deepen our understanding of the role of activation functions in deep learning but also provide valuable guidance for future model design and optimization.

### 4.3. Multimodal Data Ablation Experiment

This experiment aimed to explore and analyze the impact of using individual and combined modalities of data (image and sensor data) on the performance of the grape disease detection model, as shown in Table 4. This experimental design allowed us to clearly determine the specific contributions of different data sources to the model’s recognition capabilities and how they interact to enhance the overall detection effectiveness.

The results demonstrated significant differences in model performance when using single modal data sources (image or sensor data). With image data alone, the model exhibited higher performance with a precision of 0.90, a recall of 0.88, an accuracy of 0.89, and an mAP of 0.88. This underscores the importance of visual information in detecting grape diseases, as images provide comprehensive and detailed information about the morphology and distribution of diseases. In contrast, models relying solely on sensor data performed lower, with precision at 0.80, recall at 0.77, accuracy at 0.78, and mAP at 0.78. This may be due to sensor data providing crucial information about environmental conditions and grape physiological status, yet lacking spatial details that are essential to independently deliver comprehensive insights into specific disease types and severity. When image and sensor data were used in combination, there was a notable improvement in model performance, with precision increasing to 0.93, recall at 0.90, accuracy at 0.91, and mAP at 0.91. This result indicates that the combination of different modal data not only complements each one’s deficiencies but also enhances the model’s accuracy and robustness in identifying grape diseases. By integrating visual and environmental information, the model is enabled to understand and analyze the complexities of grape diseases more comprehensively, thus achieving more accurate disease identification in varying real-world applications.

Theoretically, and from the model’s mathematical characteristics, the advantages of data fusion can be explained through information theory and decision theory. Image data provide high-dimensional spatial information, aiding the model in capturing visual features of diseases, while sensor data offer time-series environmental and physiological information, assisting the model in understanding the environmental factors influencing disease development. Combining these allows for more comprehensive decision-making, enhancing feature expression and decision diversity, and thus improving the model’s overall performance. Additionally, multimodal fusion, by expanding the feature space and providing richer contextual information, helps the model overcome limitations of single data sources, improving adaptability and generalization capabilities towards complex disease patterns.

### 4.4. Lightweight Deployment

In contemporary research on grape disease detection, lightweight models capable of operating on mobile devices like the iPhone 15 hold significant practical value, especially given the portability and utility of mobile devices in field conditions. Therefore, our research team developed a lightweight model optimized for mobile endpoints, ensuring rapid and efficient onsite disease detection while maintaining high accuracy. Initially, the development process began with model simplification, employing structural pruning techniques to reduce the model’s complexity. This technique involves evaluating the importance of weights in each convolutional layer, where less critical weights are pruned, thereby reducing the number of model parameters and computational demands. Mathematically, the importance of weights can be calculated using the following formula:

(22) $$I\left(w\right)=\sum_{i=1}^{N}\left|w_{i}\right|$$

where $w_{i}$ represents the weights, *N* is the total number of weights, and I(w) is the importance indicator of the weights. The pruned model significantly lightens the burden on memory and processing speed, making it more suitable for devices with limited computing resources. Next, to further reduce the model’s runtime latency, quantization techniques were employed to convert the model’s floating-point weights into fixed-point numbers with lower bit-width. This process is mathematically represented as

(23) $$w_{q}=round\left(\frac{w}{scale}\right)$$

where $w_{q}$ is the quantized weight, *w* is the original weight, and scale is the quantization scale factor. Through quantization, the model’s size is further compressed, reducing runtime energy consumption and enhancing execution speed. Additionally, considering the limited GPU resources on mobile devices, the model utilizes depthwise separable convolutions instead of traditional convolutions, significantly reducing the model’s computational complexity and parameter count. Depthwise separable convolutions first apply depthwise convolution to independently process each channel of the input, followed by a pointwise convolution to combine these channel outputs. Compared to standard convolutions, this reduces computational load and model size. The specific mathematical expression is

(24) $$y_{k}=\sum_{i=1}^{M}\left(k_{i}*x_{i}\right)$$

where $k_{i}$ is the *i*-th kernel in the depthwise convolution, $x_{i}$ is the *i*-th channel of the input features, and $y_{k}$ is the output of the *k*-th channel. This method ensures that each channel is processed independently, lowering the model’s computational complexity and enhancing efficiency. To ensure smooth operation on devices like the iPhone 15, we also conducted extensive hardware-software co-optimization. By leveraging the Core ML framework, the model fully utilizes Apple’s hardware acceleration features, such as the Neural Engine (ANE). This framework provides a method for converting a trained model into a Core ML model, thereby facilitating efficient operation on iOS devices. Before deployment, the model underwent comprehensive performance evaluations, including speed tests, accuracy verification, and energy consumption analysis. Test results showed that the optimized lightweight model not only meets accuracy requirements but also achieves mobile device standards in processing speed and energy efficiency. Specifically, the model’s average response time on the iPhone 15 was reduced to less than half of the original model while maintaining an accuracy rate above 90%.

### 4.5. Scalability and Future Works

#### 4.5.1. Model Scalability

This section elaborates on the scalability of the model and its potential application strategies across various crops and plant varieties. Initially, the deep-learning-based grape disease detection model developed in this study, while primarily focused on grapes, features a highly generalizable and scalable architecture. The integration of parallel heterogeneous activation functions and multimodal data fusion techniques allows the model to efficiently process a diverse range of plant images and environmental data, not limited to grapes alone. This technological framework can be easily transferred to disease detection tasks in other crops such as wheat, corn, or other fruit trees, requiring only the substitution of the training dataset to adapt to different types of crops. Additionally, the design of our model takes into account the needs of agricultural production on varying scales. For large-scale planting scenarios, the model supports efficient data processing and analysis on cloud computing platforms, capable of handling substantial data volumes from extensive farmlands. Moreover, the lightweight design of the model also supports operation on edge devices, making it suitable for use by small-scale or individual agricultural producers. This flexible deployment capability enables our model to be extensively applied in agricultural production of all sizes, from large farms to small home gardens. Furthermore, to enhance the universality and adaptability of the model, future work will focus on the study of cross-crop learning capabilities. By incorporating more advanced transfer learning technologies, the model will be better adapted to different crops and environmental conditions. In this way, the model will not only be able to detect diseases in a variety of crops but also maintain high accuracy and robustness under varying climatic and soil conditions.

#### 4.5.2. Limits Analysis

While this study has achieved significant success in grape disease detection, showcasing excellent performance in multimodal data fusion and model light-weight deployment, it still faces certain limitations and directions for future research. Firstly, although the use of multimodal data has enhanced the robustness and accuracy of the model, the data primarily originate from specific grape cultivation regions, which may limit the assessment of the model’s generalizability. The variability in climate and cultivation conditions across different regions might affect the manifestation of diseases, necessitating the further validation of the model’s applicability across diverse grape-growing areas globally. Secondly, although the model has been light-weighted to accommodate the computational resource constraints of mobile devices, its real-time responsiveness may still be challenged in practice. Especially in scenarios where data transmission and processing speeds are critically high, the current processing speed may not meet all real-time monitoring needs. Moreover, the issue of energy consumption of the model has not been thoroughly addressed in this study, which is a critical consideration for battery-powered mobile devices.

#### 4.5.3. Future Work

In future work, an in-depth exploration of the opportunities and challenges in the field of AI-driven agricultural research and applications will be conducted [50]. Despite numerous innovations and improvements brought by the application of AI in agriculture, several challenges and limitations still persist. Primarily, the acquisition and processing of data remain significant obstacles in the application of AI in agriculture. Effective data collection, accurate data analysis, and the safeguarding of data security and privacy are crucial for enhancing the efficacy of AI applications in agriculture. Future research needs to explore more efficient data processing methods to ensure the authenticity and security of data while also considering the ethical use of such data. Moreover, while AI technology can enhance agricultural productivity and the precision of crop management, the increased dependency on technology may also pose risks. Reliance on advanced technology could make agricultural production vulnerable in the event of technological failures, thereby increasing the uncertainty of agricultural outputs. Hence, future work should not only promote AI technology but also strengthen the preservation and transmission of traditional agricultural knowledge and techniques to ensure an effective integration of technology with tradition. Finally, as the application of AI in agriculture becomes more widespread, the ethical and social impacts of artificial intelligence should also become a significant area of research. Ensuring the fairness, transparency, and inclusivity of AI technology for all farmers is a crucial issue that future research cannot overlook. Through interdisciplinary collaborative research and the establishment of appropriate regulations and standards, it is possible to support the healthy development and ethical use of AI technology in agriculture.

## 5. Conclusions

This paper primarily addresses the problem of grape disease detection, proposing a deep learning model based on multimodal data and parallel heterogeneous activation functions. By integrating image and sensor data, the model significantly enhances the accuracy and robustness of disease identification. With the rapid advancement of smart agricultural technologies, achieving efficient and accurate grape disease detection not only assists farmers in timely disease management, reducing economic losses, but also provides a scientific basis for disease control, offering substantial practical value and broad market prospects. In this study, by utilizing a variety of advanced deep learning technologies, our approach exhibits excellent performance across several key performance indicators. In disease detection experiments, our method achieved an accuracy of 91%, with precision and recall rates of 93% and 90%, respectively, significantly outperforming other comparative models such as YOLOv3, YOLOv5, DETR, TinySegformer, and Tranvolution-GAN. Moreover, through ablation experiments with different activation functions, the study further validates the effectiveness of parallel heterogeneous activation functions in enhancing model performance. The results demonstrate that the combined use of various activation functions significantly surpasses the use of a single activation function, effectively improving the overall performance of the model. Additionally, this paper explores the impact of different modal data on model performance. Ablation experiments with different modal data show that the combination of image and sensor data can capture grape disease features more comprehensively. Compared to using a single data source, this significantly improves the model’s detection precision and stability. This finding confirms the critical role of multimodal data fusion in enhancing the accuracy of grape disease detection. To meet the requirements for rapid onsite detection, this study also developed a lightweight model suitable for mobile devices, successfully deployed on smart devices such as the iPhone 15. By employing techniques such as structural pruning, quantization, and depthwise separable convolutions, the computational complexity and resource consumption of the model are significantly reduced, enabling the efficient operation on devices with limited resources. This ensures real-time performance and mobile convenience, greatly enhancing the model’s application value in actual agricultural production.

## Figures and Tables

**Table 1:** Dataset distribution.

Disease	Quantity
Powdery Mildew (Figure 1a)	1567
Anthracnose (Figure 1b)	1794
Black Rot (Figure 1c)	1891
Gray Mold (Figure 1d)	1632
White Rot (Figure 1e)	1425

**Table 2:** Disease detection experiment results.

Model	Precision	Recall	Accuracy	mAP	FPS
YOLOv3	0.80	0.77	0.78	0.79	21
YOLOv5	0.83	0.80	0.81	0.81	28
DETR	0.85	0.82	0.83	0.84	35
TinySegformer	0.88	0.85	0.86	0.86	42
Tranvolution-GAN	0.90	0.87	0.88	0.89	49
Our Method	0.93	0.90	0.91	0.91	56

**Table 3:** Ablation experiment on activation functions.

Tanh	ReLU	LeakyReLU	Sigmoid	Mish	Precision	Recall	Accuracy	mAP
✓	—	—	—	—	0.75	0.73	0.74	0.74
—	✓	—	—	—	0.73	0.71	0.72	0.72
—	—	✓	—	—	0.76	0.73	0.74	0.75
—	—	—	✓	—	0.78	0.75	0.76	0.77
—	—	—	—	✓	0.77	0.73	0.75	0.74
—	✓	✓	✓	✓	0.85	0.82	0.84	0.84
✓	—	✓	✓	✓	0.83	0.81	0.82	0.82
✓	✓	—	✓	✓	0.83	0.80	0.81	0.82
✓	✓	✓	—	✓	0.86	0.84	0.85	0.84
✓	✓	✓	✓	—	0.85	0.82	0.83	0.84
✓	✓	✓	✓	✓	0.93	0.90	0.91	0.91

**Table 4:** Multimodal data ablation experiment.

Dataset	Precision	Recall	Accuracy	mAP
Sensors Dataset	0.73	0.71	0.72	0.72
Image Dataset	0.83	0.80	0.81	0.82
Both	0.93	0.90	0.91	0.91

## Data Availability

The data presented in this study are available on request from the corresponding author.
